# The Small Protein ScrA Influences Staphylococcus aureus Virulence-Related Processes via the SaeRS System

**DOI:** 10.1128/spectrum.05255-22

**Published:** 2023-05-08

**Authors:** Marcus A. Wittekind, Paul Briaud, Jayanna L. Smith, Julia R. Tennant, Ronan K. Carroll

**Affiliations:** a Department of Biological Sciences, Ohio University, Athens, Ohio, USA; University of Florida

**Keywords:** SaeRS, ScrA, *Staphylococcus aureus*, clumping, gene regulation, hemolysis, two-component system, virulence

## Abstract

Staphylococcus aureus is a Gram-positive commensal and opportunistic pathogen able to cause diseases ranging from mild skin infections to life-threatening endocarditis and toxic shock syndrome. The ability to cause such an array of diseases is due to the complex S. aureus regulatory network controlling an assortment of virulence factors, including adhesins, hemolysins, proteases, and lipases. This regulatory network is controlled by both protein and RNA elements. We previously identified a novel regulatory protein called ScrA, which, when overexpressed, leads to the increased activity and expression of the SaeRS regulon. In this study, we further explore the role of ScrA and examine the consequences to the bacterial cell of *scrA* gene disruption. These results demonstrate that *scrA* is required for several virulence-related processes, and in many cases, the phenotypes of the *scrA* mutant are inverse to those observed in cells overexpressing ScrA. Interestingly, while the majority of ScrA-mediated phenotypes appear to rely on the SaeRS system, our results also indicate that ScrA may also act independently of SaeRS when regulating hemolytic activity. Finally, using a murine model of infection, we demonstrate that *scrA* is required for virulence, potentially in an organ-specific manner.

**IMPORTANCE** Staphylococcus aureus is the cause of several potentially life-threatening infections. An assortment of toxins and virulence factors allows such a wide range of infections. However, an assortment of toxins or virulence factors requires complex regulation to control expression under all of the different conditions encountered by the bacterium. Understanding the intricate web of regulatory systems allows the development of novel approaches to combat S. aureus infections. Here, we have shown that the small protein ScrA, which was previously identified by our laboratory, influences several virulence-related functions through the SaeRS global regulatory system. These findings add ScrA to the growing list of virulence regulators in S. aureus.

## INTRODUCTION

Staphylococcus aureus is a Gram-positive commensal bacterium responsible for a wide variety of human diseases ranging from skin and soft tissue infections to life-threatening endocarditis and bacterial septicemia (1). The ability of S. aureus to infect a variety of human tissues is due to an extensive array of virulence factors, including hemolysins, adhesins, proteases, and lipases. The regulation of these virulence factor genes is mediated by a complex network of regulators comprised of both RNA- and protein-based systems. Among these regulatory elements are two-component signal transduction systems (TCSs). S. aureus encodes 16 TCSs, which respond to a variety of activation signals and regulate several key biological processes (2–6). Of the 16 TCSs present in S. aureus, several have been implicated in the regulation of virulence genes, including the Agr, Arl, and Sae systems (7–13). Of particular interest to us is the SaeRS system, which controls the expression of secreted virulence determinants, including hemolysins such as *hlgABC* and *hla*, proteases such as aureolysin, the toxic shock syndrome toxin, and immune evasion components such as nucleases and lipases. The disruption of the SaeRS system leads to the reduced production of secreted virulence factors (10, 14, 15), attenuation of virulence (16–18), and impaired biofilm development (4, 18–21).

The SaeRS system includes the SaeS sensor kinase, which has been postulated to act as an intramembrane sensor (22, 23), and the cytoplasmic SaeR response regulator. SaeR target genes have two well-defined promoters, termed class I and class II, composed of varying repeats of a conserved binding sequence (2, 16). The Sae system also consists of the accessory regulators SaeP and SaeQ, which act to modulate the system by influencing SaeS phosphatase activity. Consequently, SaePQ play an important role in returning the system to a prestimulation state (2, 19, 24). Although SaePQ are known to induce phosphatase activity in SaeS, little else is known about the proteins, including potential alternative interaction partners.

We previously identified a positive regulator of the Sae system, the small protein ScrA, which increases Sae activity through an undetermined mechanism (25). The overexpression of *scrA* led to (i) the rapid agglutination of bacterial cells, (ii) decreased membrane stability, and (iii) an increase in the hemolytic activity of the culture supernatants (all in an Sae-dependent manner). Although our previous study described these phenotypes, the specific bacterial virulence factors underlying them were not identified. Furthermore, our previous study relied exclusively on *scrA* overexpression, and the effect of an *scrA* mutant was not investigated. In this study, we sought to identify the specific toxins responsible for the previously identified increase in hemolytic activity and decreased membrane stability (25). Additionally, we have characterized an *scrA* mutant and demonstrated that the loss of ScrA leads to defects in aggregation in the presence of human serum and a reduction in dissemination to the heart in a murine model of systemic infection. Collectively, these results build on those of our previous study and demonstrate that ScrA contributes to S. aureus pathogenesis *in vivo*.

## RESULTS

### ScrA influences cellular aggregation differentially in the presence of host factors.

**FIG 1:**
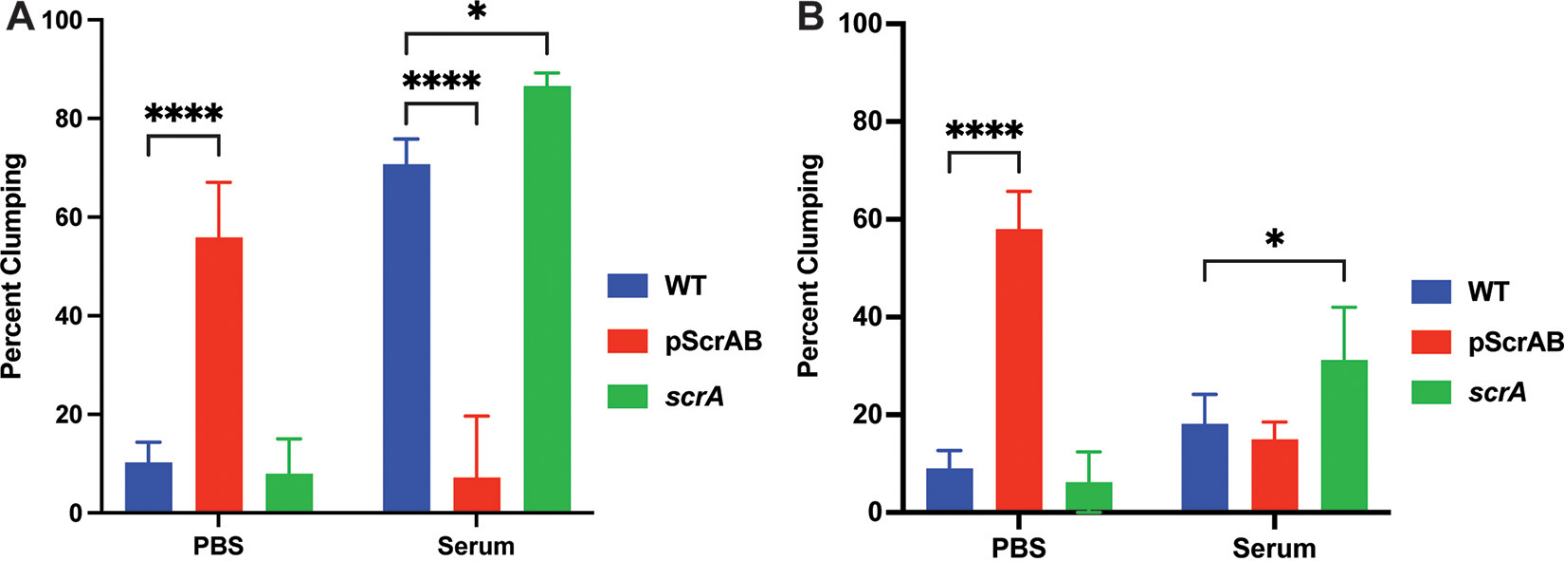
Rotating (A) and static (B) clumping assays of ScrA-overexpressing and *scrA* mutant strains in PBS and human serum. (A) Cultures were incubated with rotation in 600 mL whole human serum for 30 min. After incubation statically at room temperature for 5 min to allow clumps to settle, the OD_600_ of the top 100 mL was determined. (B) Rotating cultures were resuspended by pipetting and vortexing to disperse aggregates and incubated statically at room temperature for 40 min. The OD_600_ of the top 100 mL was determined. The reduction in the OD_600_ from that of the initial culture is indicative of clumping. Statistical significance was determined by two-way analysis of variance (ANOVA). *, *P* < 0.5; ****, *P* < 0.001.

Our previous work demonstrated that ScrA overexpression led to increased cellular aggregation in statically incubated, planktonically grown cultures (25). However, previous work also demonstrated that the expression level of *scrA* (previously named *tsr37* or SAUSA300s301) is ~16-fold lower in cells grown in human serum than in those grown in tryptic soy broth (TSB) (26). The relative abundance of large and small S. aureus surface adhesins has been shown to control intercellular aggregation and host factor-mediated clumping (27). Since ScrA appears to modulate SaeRS activity (25), and *scrA* expression is influenced by human serum (26), we hypothesized that the abundance of ScrA within a cell would influence binding to host factors by altering the relative abundance of surface proteins. To investigate if ScrA influences binding to host factors, we performed a modified clumping assay. We have observed that ScrA-induced aggregates can be readily dispersed by agitation, so to minimize cellular aggregation, we performed a clumping assay while rotating the samples (see Fig. S1A in the supplemental material). After 60 min of rotating incubation, samples were incubated statically for 5 min, allowing aggregates to settle to the bottom of the tubes. Samples rotated in this assay in phosphate-buffered saline (PBS) demonstrated an increase in cellular aggregation when ScrA was overexpressed (similar to our previous findings), while no difference was observed between the wild type (WT) and the *scrA* mutant (Fig. 1A). Interestingly, when cells were rotated in human serum, the opposite trend was observed, with the ScrA-overexpressing strain showing a decrease in clumping relative to the empty vector controls. Furthermore, the *scrA* mutant demonstrated a slight (but significant) increase in clumping relative to both the ScrA-overexpressing strain and the WT (Fig. 1A).

Next, the samples described above were resuspended by vortexing (to break up previously formed clumps), the top 100 μL was removed, and the optical density at 600 nm (OD_600_) was determined. The cultures were then incubated statically at room temperature for 40 min, the top 100 μL was removed, and the OD_600_ was measured. The difference in the OD_600_ values before and after the 40-min incubation was expressed as percent clumping (Fig. 1B). The results for the PBS samples were consistent with those with rotating incubation (Fig. 1A); however, unlike the samples in the original assay (Fig. 1A), the ScrA overexpression strain in serum now showed no significant decrease in aggregation compared to the WT. These phenotypes are likely due to changes in proteins involved in intracellular binding as opposed to proteins involved in host factor binding in the overexpression strain. It is known that larger surface adhesins may interfere with host factor and intercellular binding (27). Likely, the deletion of *scrA* leads to the decreased expression of large surface proteins, which allows more efficient binding to host factors. This reduction in large surface proteins would reduce interference and allow host factors to move within the binding range of the smaller adhesins residing closer to the cell. This raises the question of which host factors are being bound, and which larger S. aureus surface proteins are absent when *scrA* is deleted.

### ScrA influences host factor binding and surface protein expression.

To investigate if host factor binding was being altered by the presence of *scrA*, we performed a cell wall “shaving” assay on cells incubated in either PBS or human serum. Bacterial cells were incubated with either PBS or human serum while rotating before being washed. Surface proteins (and any associated host proteins) were then removed by incubating cells with immobilized trypsin, after which cells were pelleted and the supernatant was collected. The supernatants, containing extracted protein fragments, were analyzed by mass spectrometry (MS).

**FIG 2:**
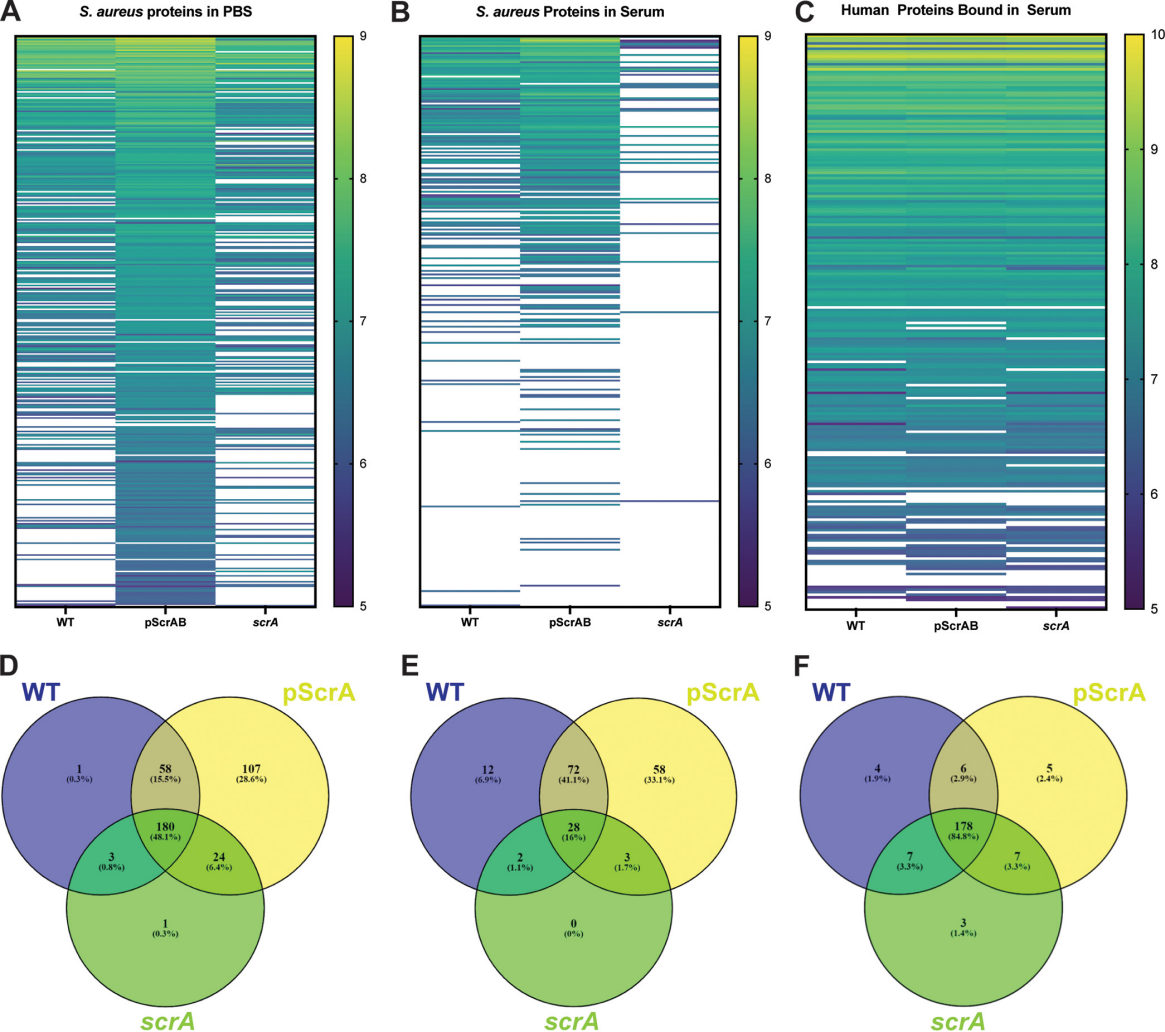
S. aureus and human proteins identified by cell wall shaving assays. Bacterial cells were incubated in either PBS or whole human serum. (A and D) S. aureus proteins identified from cells incubated in PBS. (B and E) S. aureus proteins identified from cells incubated in serum. (C and F) Human proteins identified from cells incubated in PBS. Heat maps (A to C) represent proteins identified in the indicated strains, and the data shown are the averages from triplicate samples. Venn diagrams (D to F) indicate the number of proteins uniquely identified in each strain, the number of proteins found in two out of three strains, and the number of proteins found in all three strains.

The mass spectrometry results were grouped into 3 categories, S. aureus proteins identified from samples where S. aureus was incubated in PBS (Fig. 2A and D and Data Set S1), S. aureus proteins identified from samples where S. aureus was incubated in human serum (Fig. 2B and E and Data Set S2), and human proteins identified from samples where S. aureus was incubated in serum (Fig. 2C and F and Data Set S3). Within the S. aureus proteins identified from samples incubated in PBS (Fig. 2A), several proteins were identified only in ScrAB-overexpressing strains. Of note, several of the 107 unique proteins found in the ScrAB overexpression strain in PBS (Fig. 2D) are predicted cytoplasmic proteins. Previous work from our group showed that ScrAB overexpression leads to decreased membrane stability (25), which likely accounts for the large number of cytoplasmic proteins identified in these samples. This would explain the decreased number of S. aureus proteins detected in the wild-type and *scrA* mutant strains incubated in PBS. Only 1 protein (nuclease) was uniquely identified in the wild-type strain, and only 1 protein (SAUSA300_2097) was uniquely identified in the *scrA* mutant strain (Fig. 2D). Of the 107 proteins uniquely identified in the ScrAB-overexpressing strain, 7 of the top 10 most abundant proteins are predicted cytoplasmic proteins (Table 1 and Data Set S1).

**TABLE 1:** S. aureus proteins uniquely identified in samples incubated in PBS

Gene product	Function
Unique in the WT	
Nuc	Secreted nuclease
Unique in the ScrA overexpressor (10 most abundant)	
Emp	Surface adhesin
LukS	Toxin
FnbA	Surface adhesin
Ear	Secreted protein Ear
RpsB	Ribosome component
TarJ	Ribitol-5-phosphate dehydrogenase
RplF	Ribosome component
RplV	Ribosome component
PyrC	Dihydro-orotase
SelX	Enterotoxin-like toxin X
Unique in the *scrA* mutant	
SAUSA300_2097	Hypothetical protein

S. aureus proteins identified from samples incubated in human serum (Fig. 2B) showed a trend that was overall similar to that of samples incubated in PBS (Fig. 2A); i.e., more proteins were uniquely identified from samples of the ScrAB-overexpressing strain. In total, 12 proteins were uniquely identified in wild-type samples, 58 proteins were uniquely identified in the ScrAB-overexpressing samples, and 0 unique proteins were identified from the *scrA* mutant (Fig. 2E). Interestingly, only 33 total proteins were identified in *scrA* mutant samples. This could be explained by the increased clumping observed in the *scrA* mutant incubated in human serum (Fig. 1B), which may prevent trypsin from accessing surface proteins. Once again, several predicted cytoplasmic proteins were uniquely identified in both the wild-type and ScrAB-overexpressing samples (Table 2 and Data Set S2).

**TABLE 2:** S. aureus proteins uniquely identified in samples incubated in serum

Gene product	Function
Unique in the WT	
SAUSA300_0555	Metabolism
SAUSA300_0307	Metabolism
SAUSA300_0173	Hypothetical protein
RpsP	Ribosomal component
RplO	Ribosomal component
SAUSA300_1582	Hypothetical protein
RpsO	Ribosomal component
SAUSA300_1685	Hypothetical protein
SAUSA300_1491	Metabolism
SAUSA300_1698	Hypothetical protein
Mqo	Metabolism
FloA	Hypothetical protein
Unique in the ScrA overexpressor (10 most abundant)	
Coa	Adhesin
FnbA	Surface adhesin
Emp	Surface adhesin
RpsM	Ribosomal component
RplK	Ribosomal component
Ear	Hypothetical protein
Hpf	Ribosomal component
FruA	Metabolism
LukF	Toxin
RplB	Ribosomal component

Finally, we looked at human proteins bound by S. aureus cells incubated in human serum. Overall, there were few differences in human proteins bound by S. aureus cells (Fig. 2C), with the wild-type, ScrA overexpressor, and *scrA* mutant strains having 4, 5, and 3 unique proteins, respectively (Fig. 2F, Table 3, and Data Set S3). The unique proteins from the wild-type strain were all identified as human immunoglobulin components, while the unique proteins from the ScrA-overexpressing strain included extracellular matrix and cell structure proteins. This is unsurprising as the extracellular matrix binding protein Emp was one of the unique proteins identified from ScrA-overexpressing samples incubated in PBS and human serum (Tables 1 and 2). Unfortunately, none of the 3 unique proteins identified in the *scrA* mutant strain could explain the increased clumping observed in this strain, with 2 proteins being immunoglobulin components and 1 being a sulfhydryl oxidase (Table 3). Interestingly, 2 coagulation factors, X and XIII, were identified in the wild-type and overexpressing strains but not in samples of the *scrA* mutant.

**FIG 3:**
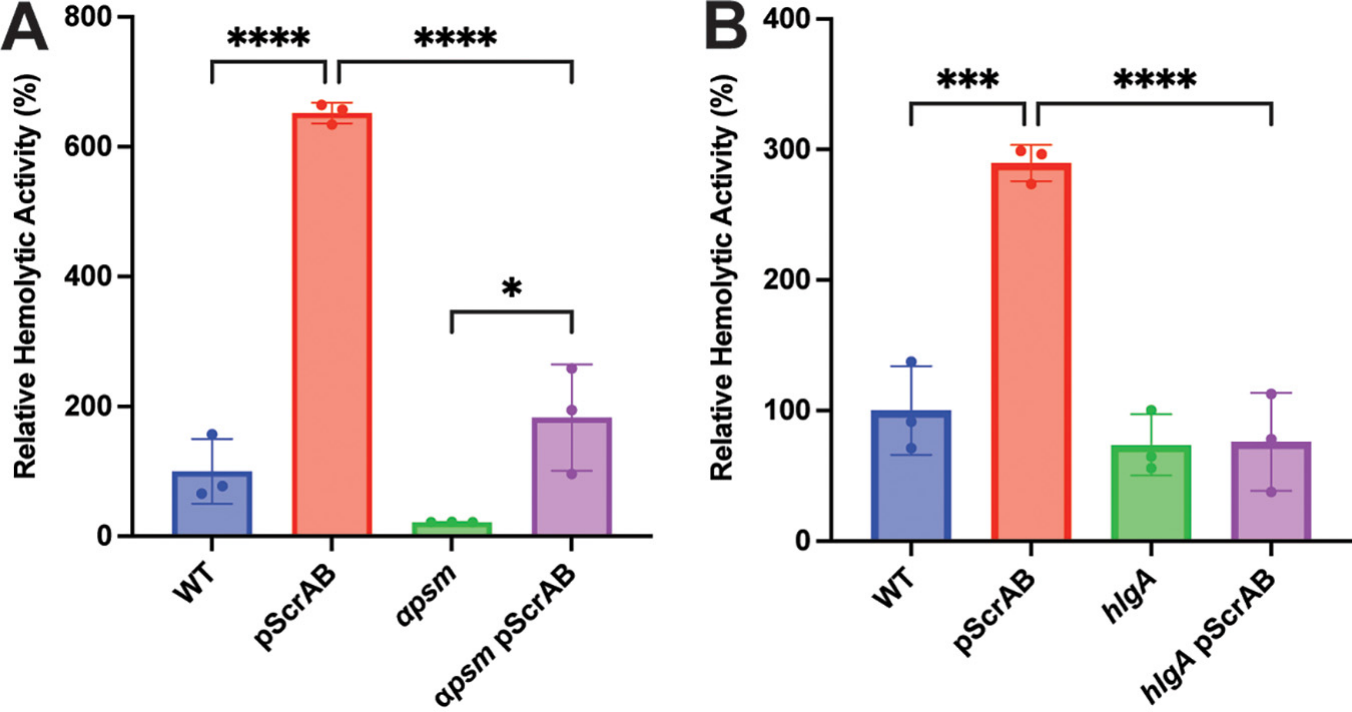
ScrA-induced hemolysis is dependent on S. aureus gamma-hemolysin. (A) ScrA overexpression in an αPSM mutant results in an increase in hemolysis over the empty vector control. (B) ScrA overexpression in an *hlgA* mutant does not lead to an increase in hemolytic activity. Statistical significance was determined using a one way ANOVA with Tukeys multiple comparison. *, *P* < 0.5; ***, *P* < 0.005; ****, *P* < 0.001.

**TABLE 3:** Human proteins uniquely identified in samples incubated in serum

Gene product	Function
Unique in the WT	
IGKV1D-8	Immunoglobulin component
IGHV3OR15-7	Immunoglobulin component
IGHV1-2	Immunoglobulin component
IGHV4-4	Immunoglobulin component
Unique in the ScrA overexpressor	
ECM1	Extracellular matrix protein
DSG1	Desmoglein 1
CD14	Pattern recognition receptor
FLNA	Cell structure protein
TLN1	Cell structural protein
Unique in the *scrA* mutant	
IGHV3-23	Immunoglobulin component
IGHV2-26	Immunoglobulin component
QSOX1	Sulfhydryl oxidase
Common to the WT and the ScrA overexpressor	
IGHD	Immunoglobulin component
F13A1	Coagulation factor XIII
IGLV2-18	Immunoglobulin component
IGLV9-49	Immunoglobulin component
F10	Coagulation factor X
ADIPOQ	Adiponectin

When comparing the relative abundances of proteins, we identified some notable differences. In the *scrA* mutant incubated in PBS, the level of the hypothetical protein SAUSA300_0602 was increased 1.85-fold over that of the wild type and 10.03-fold over that of the ScrAB overexpression strain (Table 4 and Data Set S1). The level of ClfA was increased 4.25-fold over that of the ScrAB overexpressor, while both FnbA and Efb were not present in the *scrA* mutant (Table 4). This increase in ClfA may result in increased binding to von Willebrand factor (vWf), as it has been shown previously that von Willebrand binding factor produced by S. aureus facilitates ClfA and vWf binding (28). In human serum, we observed a 6.25-fold increase in vWf bound by the *scrA* mutant compared to the wild type (Table 4 and Data Set S2). However, the ScrAB-overexpressing strain demonstrated a 47-fold increase in bound von Willebrand factor over that of the wild type and a 7.64-fold increase in bound von Willebrand factor over that of the *scrA* mutant (Table 4). This is unsurprising as ScrAB overexpression was previously shown to increase the expression of von Willebrand binding factor (25). Additionally, several host proteins bound by the *scrA* mutant, including coagulation factor XIII, extracellular matrix proteins, and filamin A, showed either an insignificant change compared to wild type or a significant decrease compared to the ScrAB overexpressor (Table 4 and Data Set S3). Interestingly, fibrinogen bound by the *scrA* mutant was increased 1.66-fold compared to the wild type and 1.92-fold compared to the ScrAB overexpressor (Table 4). This increase in fibrinogen binding occurs despite an apparent decrease in Efb, with the *scrA* mutant expressing a 2.85-fold-lower level than the wild type and a 10.59-fold-lower level than the ScrAB overexpressor. This apparent discrepancy may again be due to the clumping observed in the *scrA* mutant. Increased binding to host factors may result in the decreased efficacy of cell wall shaving by trypsin through blocking access to proteins. Another possible explanation is the activity of polysaccharide intracellular adhesin (PIA), which would not be determined by the mass spectrometry experiment.

**TABLE 4:** Select proteins showing statistically significant differential abundances by proteomic analysis^*a*^

Gene product or function	Organism	Fold change		
		pScrAB/EV	*scrA*/EV	*scrA*/pScrAB
PBS				
SAUSA300_0602	S. aureus	NS	1.85	10.03
ClfA	S. aureus	NS	NS	4.25
FnbA	S. aureus	∞	NS	−∞
Efb	S. aureus	1.41	−∞	−∞
Serum				
Fibrinogen	Human	NS	1.66	1.92
von Willebrand factor	Human	47.74	6.25	−7.64
Coagulation factor XIII	Human	26.18	NS	−∞
Extracellular matrix protein	Human	∞	NS	−∞
Filamin A	Human	∞	NS	−∞
Efb	S. aureus	3.72	−2.85	−10.59

a ∞, not identified in the denominator sample; −∞, not identified in the numerator sample; EV, empty vector; NS, not significant. Fold change values are only displayed for proteins demonstrating statistically significant differences. Significance was determined by Students t-test.

No single protein appears to be responsible for the clumping observed in the *scrA* mutant. Therefore, it is likely that, rather than a single factor, the observed clumping is due to the dysregulation of several surface proteins and potential activity by nonprotein adhesion mechanisms such as PIA.

### ScrA-induced hemolysis is mediated by HlgAB.

The overexpression of ScrA was previously shown to increase the hemolytic activity of S. aureus culture supernatants (25); however, we did not previously investigate the specific toxin(s) responsible for this increase. Previous work by us and others (29–32) demonstrated that the phenol-soluble modulins (PSMs) are potent at lysing human erythrocytes. To investigate if PSMs were responsible for the observed increase in hemolysis, we performed an erythrocyte hemolysis assay using cell-free culture supernatants from WT S. aureus and an αPSM mutant strain containing either the ScrA overexpression plasmid or an empty vector control. As previously demonstrated, the overexpression of ScrA leads to the increased hemolysis of WT S. aureus (Fig. 3A). Overall, the hemolysis levels were reduced in the αPSM mutant compared to the wild type; however, there was still a significant increase in hemolysis when ScrA was overexpressed in the αPSM mutant (Fig. 3A). This result suggests that while the αPSMs contribute to erythrocyte hemolysis, another toxin is responsible for the increase observed upon ScrA overexpression.

Proteomics analysis of the secreted fraction of an ScrA-overexpressing strain showed an increased abundance of gamma-hemolysin components (25). It was previously shown that the bivalent toxin HlgAB also has cytolytic activity toward human erythrocytes (15, 33). To determine if ScrA overexpression leads to increased hemolysis through Hlg overproduction, we performed a hemolysis assay by overexpressing ScrA in an *hlgA* mutant background and comparing the results to those for the empty vector controls. Once again, in the WT strain, the overexpression of ScrA led to increased hemolysis; however, when ScrA was overexpressed in an *hlgA* mutant, no increase in hemolytic activity was observed (Fig. 3B). This result strongly suggests that HlgA is mediating the ScrA-dependent increase in hemolytic activity.

### ScrA-mediated hemolytic activity requires the SaeRS system.

**FIG 4:**
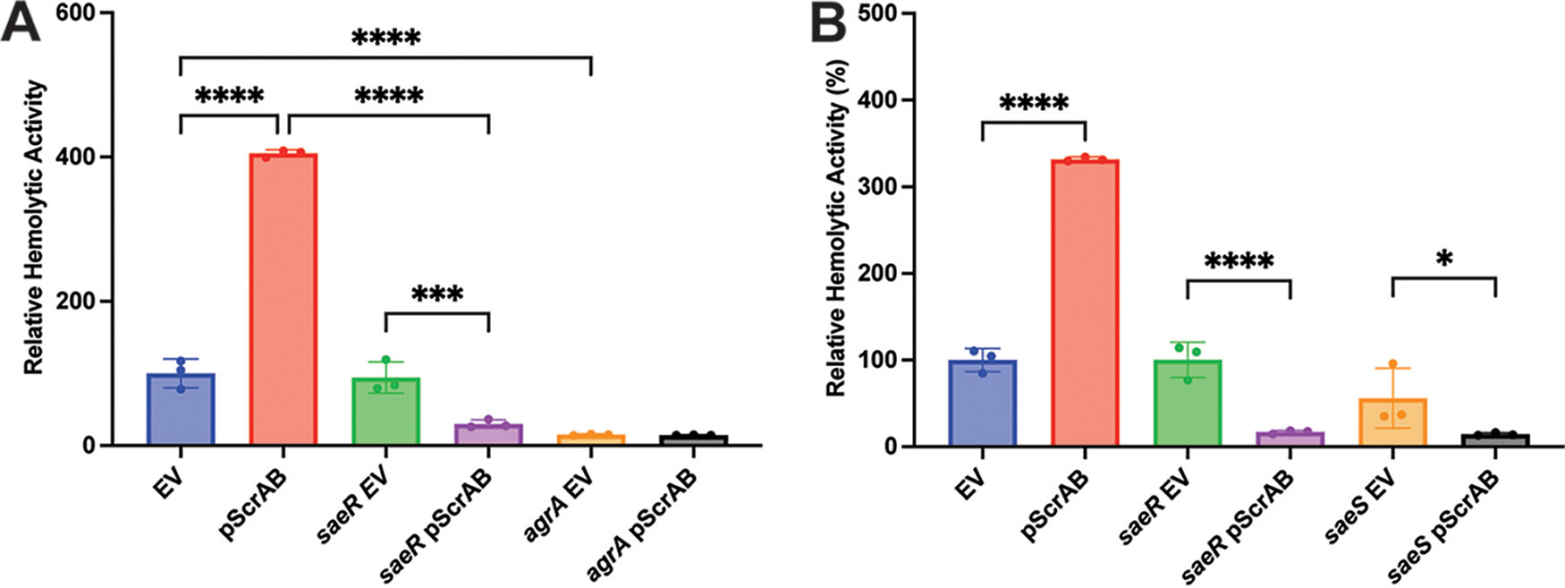
ScrA overexpression leads to reduced hemolysis in an Sae mutant. (A) ScrAB overexpression leads to a decrease in hemolytic activity in an *saeR* mutant. No change in hemolytic activity is observed upon ScrA overexpression in an *agrA* mutant. (B) Overexpression of ScrA in either an *saeR* or an *saeS* mutant background leads to decreased hemolytic activity. *, *P* < 0.5; ***, *P* < 0.005; ****, *P* < 0.001.

While we previously established that ScrA-mediated cellular aggregation was dependent on the SaeRS system, we also wanted to determine if this requirement extended to the increase in hemolysis. To investigate this, we overexpressed ScrA in an *saeR* mutant strain and quantified the hemolytic activity. Surprisingly, we observed a decrease in the hemolytic activity when ScrA was overexpressed in an *saeR* mutant, compared to the empty vector control (Fig. 4A). To investigate if the decrease in hemolysis observed in the *saeR* mutant was due to aberrant SaeS activity, we next overexpressed ScrA in an *saeS* mutant. A similar reduction in hemolytic activity was observed following the overexpression of ScrA in the *saeS* mutant background (Fig. 4B). This suggests that in addition to activating the Sae system, there may be an additional unidentified target/role for ScrA.

### ScrA-mediated hemolysis requires the Agr system.

In addition to investigating the role of the Sae system in ScrA-mediated hemolysis, we investigated if the quorum-sensing Agr system is required for the ScrA-mediated increase in hemolytic activity. It is well established that the Agr system influences the expression of hemolysin genes and is also known to regulate *sae* transcription (8, 34, 35). As such, it constitutes a potential alternative target for ScrA. The overexpression of ScrA in an *agrA* mutant resulted in no increase in hemolytic activity (Fig. 4A), suggesting that ScrA-induced hemolysin production requires a functional Agr system. However, it remains unclear if this is due to a direct interaction between ScrA and Agr or is indirect, possibly due to Agr activity on the Sae system.

### ScrA is not essential for hemolysis.

**FIG 5:**
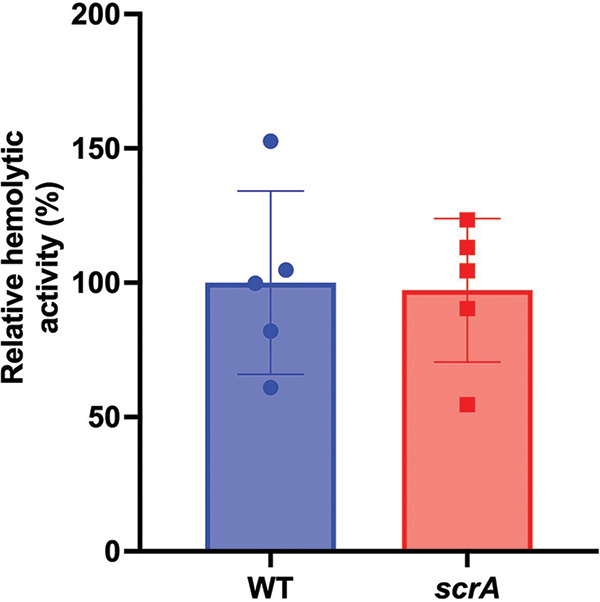
ScrA is not required for hemolysis in S. aureus. A hemolysis assay was performed using wild-type S. aureus and an *scrA* mutant strain. No significant difference in hemolysis was observed in the mutant strain.

While we have demonstrated that the overexpression of ScrA leads to increased hemolytic activity via Hlg, we have yet to determine if ScrA is essential for hemolysis. To investigate this, we performed a cell-free hemolysis assay using the wild-type and *scrA* mutant strains. We observed no significant change in hemolytic activity between the wild type and the *scrA* mutant (Fig. 5), suggesting that while ScrA overexpression can cause an increase in hemolytic activity, it is not required for hemolysis in the wild-type background.

### ScrA stabilizes the S. aureus membrane potentially by regulating lipase activity.

**FIG 6:**
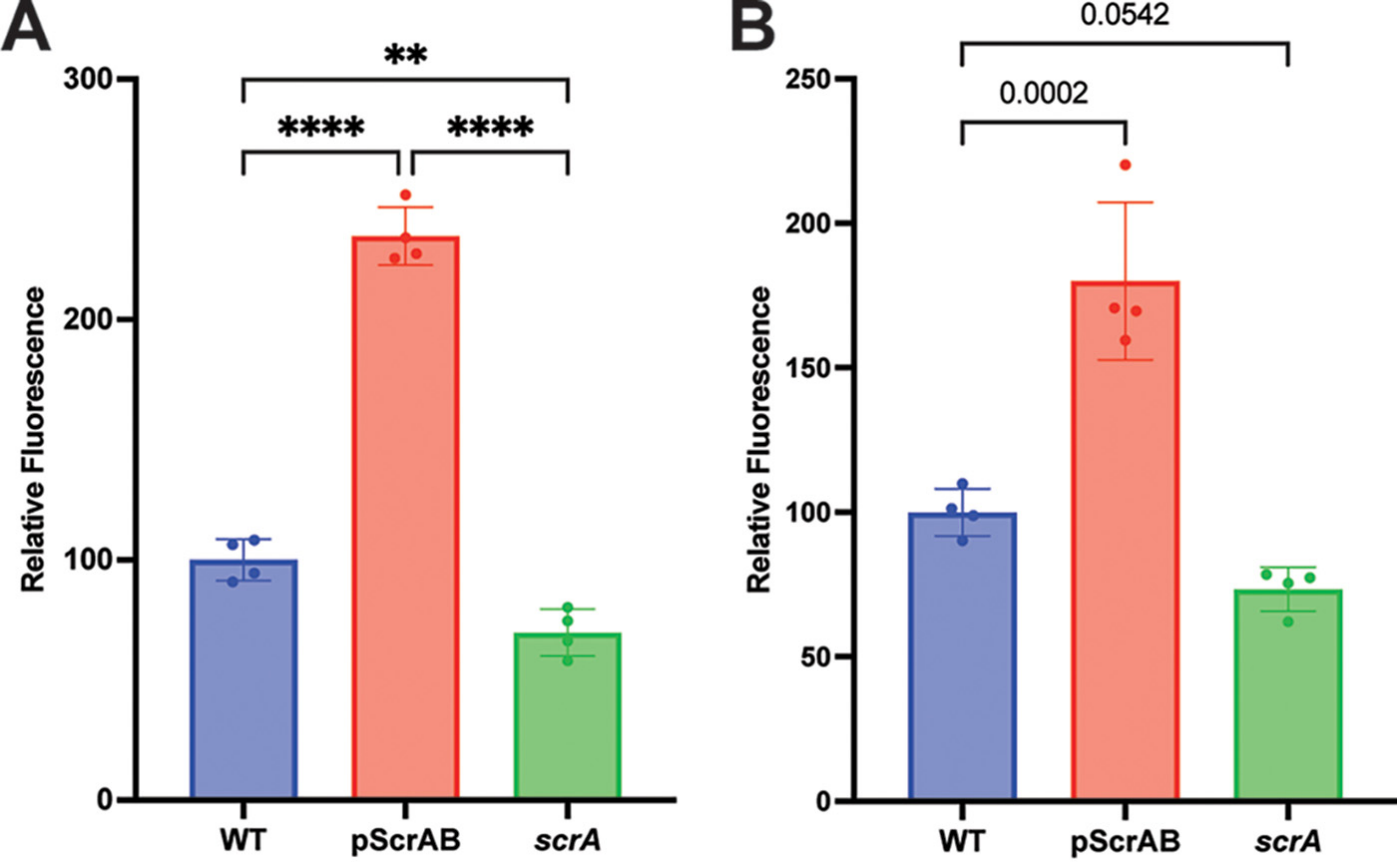
Membrane stability and lipase activity are similarly impacted by ScrA. Membrane stability (A) and lipase activity (B) were determined in ScrA-overexpressing and *scrA* mutant strains. (A) ScrA-overexpressing strains show increased membrane instability, while an *scrA* mutant shows increased membrane stability. (B) Lipase activity mirrors membrane stability, with the ScrA-overexpressing strain demonstrating increased lipase activity and the *scrA* mutant demonstrating a modest, although nonsignificant (*P* = 0.0542), decrease in lipase activity. *, *P* < 0.5; ****, *P* < 0.001.

A decrease in membrane stability was previously observed when ScrA was overexpressed, and the instability was shown to be dependent on the presence of SaeRS (25). We hypothesized this was due to a downstream effect mediated by a member of the SaeRS regulon. To further investigate this phenotype, we first determined if the opposite effect occurred in an *scrA* mutant strain. To do so, we once again employed a propidium iodide staining assay. Propidium iodide is unable to pass intact membranes, and therefore, an increase in staining indicates membrane instability and penetration of the dye into the cell. The results demonstrated a slight increase in membrane stability in the *scrA* mutant strain (Fig. 6A). Membrane stability was decreased following the overexpression of ScrA (25).

Potential mediators of this membrane instability are staphylococcal lipases, which are influenced by SaeRS (4). Some lipases, such as S. aureus lipase 3 (SAL3), have been shown to bind to phosphatidylglycerol, which is abundant in the S. aureus membrane (36). A change in the lipase abundance may be sufficient to influence S. aureus membrane stability. To investigate if there is a difference in lipase activity, we utilized the QuantiChrom lipase activity kit, used in previous studies of S. aureus lipases (36). We observed a significant increase in lipase activity in the ScrA overexpression strain (Fig. 6B), consistent with the increase in propidium iodide staining/membrane instability. Similarly, a reduction in lipase activity was observed in the *scrA* mutant, which mirrored the trend observed in the propidium iodide staining assay (although the reduction in lipase activity was deemed nonsignificant [*P* = 0.0542]). Due to the consistency between the results of the lipase activity and membrane stability assays, we consider it likely that an altered lipase abundance affects membrane stability; however, these results are correlative at this point and do not establish a direct link between altered lipase activity and membrane stability.

### ScrA is required for full virulence in a murine model of systemic infection.

**FIG 7:**
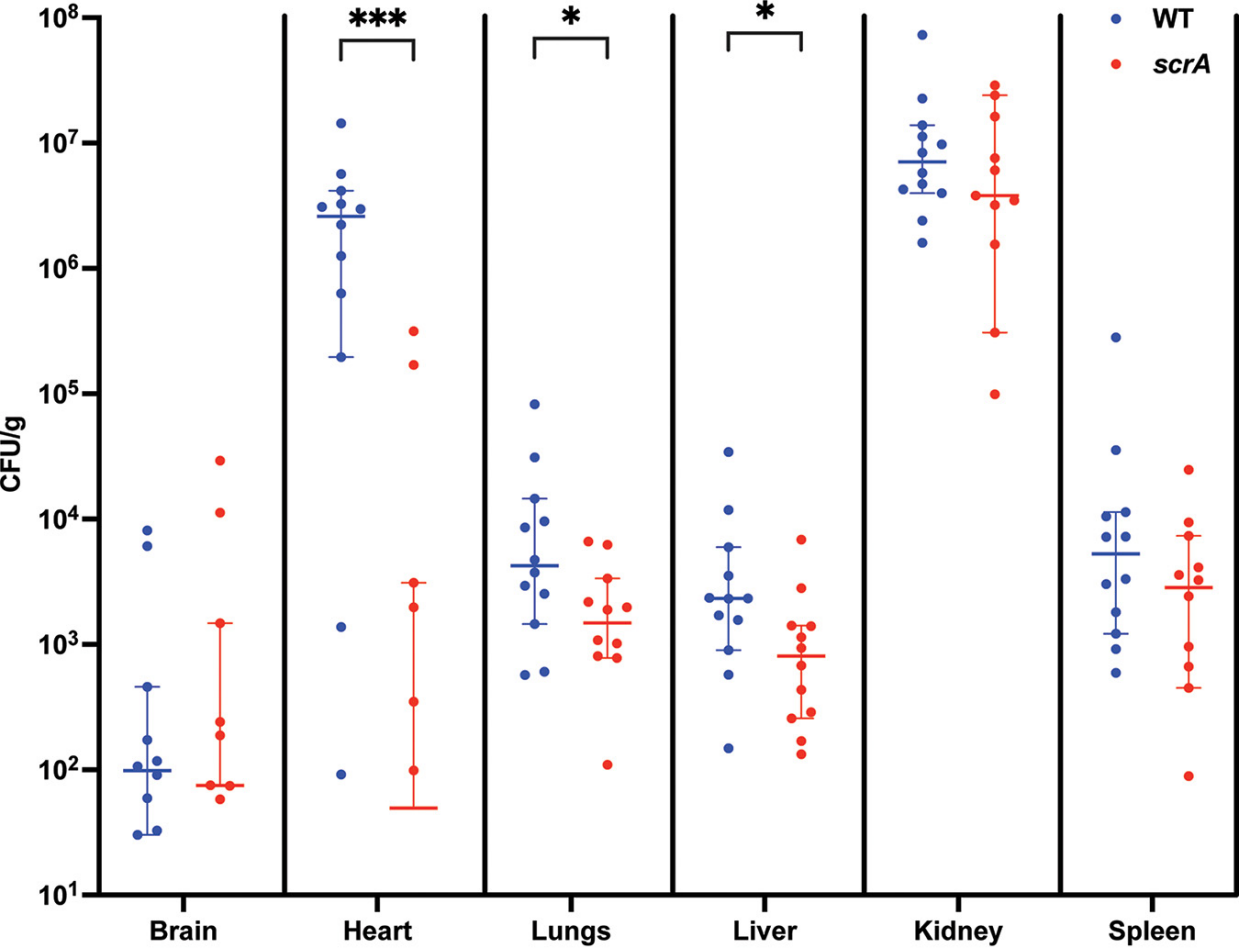
Murine model of systemic infection. Mice were infected with either the WT or the *scrA* mutant strain. Infection progressed for 3 days before euthanasia and organ harvest. The bacterial burdens in the brain, heart, lungs, liver, kidneys, and spleen were determined. Significant decreases in the bacterial burdens in the heart, lungs, and liver were observed, with the heart showing the largest decrease in the bacterial burden. Significance was determined using a Mann-Whitney test. *, *P* < 0.5; ***, *P* < 0.005.

While we were able to identify several virulence-related phenotypes following *scrA* inactivation or overexpression *in vitro*, it remained unclear if these would culminate in a biologically relevant change in virulence *in vivo*. Therefore, to investigate this, we performed a murine model of systemic dissemination of infection using wild-type and *scrA* mutant strains of S. aureus. Groups of 12 mice were inoculated, and after 3 days, the bacterial burdens in the brain, heart, lungs, liver, kidneys, and spleen were determined. Compared to mice infected with the wild-type strain, *scrA* mutant-infected mice had decreased bacterial burdens in the heart, lungs, and liver (Fig. 7), while there was no significant difference in the brain, kidneys, or spleen. It is worth noting that the decrease in the bacterial burden in the heart is ~2 logs higher than the reduction observed in the lungs or liver, with mean differences of −3.11 × 10^6^ (heart), −1.14 × 10^4^ (lungs), and −1.01 × 10^4^ (liver). This striking difference for the *scrA* mutant in the heart is suggestive of an organ-specific role for ScrA during infections. Overall, these data show that *scrA* is required for the full virulence of S. aureus.

## DISCUSSION

The regulation of S. aureus virulence is a complex process requiring strict control by both RNA and protein systems. Two-component systems represent one part of this regulatory network and are canonically thought of as environmental sensors. Accessory proteins that modulate the activity of these two-component systems are well established in S. aureus (3, 19, 24, 37). We previously identified the small protein ScrA as a potential regulator of the SaeRS system; however, our initial investigation relied exclusively on the overexpression of ScrA, which resulted in a variety of pronounced phenotypes (25). In this study, we show that the phenotypes in an *scrA* mutant strain are typically inverse to those of an ScrA overexpressor and, in general, are more subtle. The subtlety of these phenotypes, in addition to the relatively low basal levels of *scrA* expression, suggests that ScrA may be expressed and required only in a specific niche. Murine infection models corroborate this hypothesis due to the marked decrease in the bacterial burden observed in mice infected with the *scrA* mutant, particularly in the heart. Previously, we have shown that von Willebrand binding factor is >100-fold more abundant in the secretome when ScrA is overexpressed (25). Additionally, in this study, we observed a 47-fold increase in bound von Willebrand factor (Table 4). It has been established that S. aureus binding to von Willebrand factor allows adherence to endothelial tissue and resistance to sheer forces, which are essential for the early stages of endocarditis (38, 39). It is conceivable that in the absence of ScrA, S. aureus cells have a reduced ability to adhere to the heart, which results in a reduction in the bacterial burden.

However, the array of environments experienced by the bacterium during *in vivo* infection makes it difficult to know the precise conditions that stimulate *scrA* expression. It is tempting to speculate that ScrA may play a role in neutrophil interactions as many of the conditions encountered during interactions with neutrophils, such as human neutrophil peptide 1, H_2_O_2_, and decreased pH, are known to stimulate SaeS (2, 9, 17, 40, 41). It is possible that under normal conditions, ScrA expression would reduce the fitness of the cell, which may explain the strict regulatory control over *scrA*. As it stands, however, more study is needed to determine the exact environment and signals that stimulate *scrA* expression.

Cellular aggregation is a well-established immune evasion strategy used by bacteria to prevent phagocytosis (42). However, release from these aggregates is equally important, and for S. aureus, this is mediated by proteins such as staphylokinase and the binding of plasminogen (42–44). Additionally, release from biofilms during the exodus phase, mediated by proteases and nucleases, is required for the dissemination of the bacteria to distal sites (45). For optimal fitness, S. aureus must balance clumping and dispersal. ScrA may act at low levels to modulate the activity of SaeS to maintain an equilibrium in this binding-and-release model. This hypothesis is extended to include the regulation of lipases, which are correlated with membrane stability in our study (Fig. 6). Bacterial membranes represent a complex fluid mosaic that is in a constant state of change, and the presence of microdomains appears to play an essential role in bacteria (46–48). The previously reported protein SAL3 has been shown to have a binding affinity for lipids found within the S. aureus membrane (36). Our data suggest that in addition to the dysregulation of adhesins, alterations in the ScrA abundance also lead to dysregulated lipase expression/activity, which, when paired with the observed membrane stability changes, leads us to hypothesize a role for ScrA in regulating membrane stability through lipase activity.

Previous work by our laboratory had demonstrated that ScrA-mediated cellular aggregation and membrane instability required the presence of a functional SaeRS system. This strongly suggested a direct interaction between ScrA and some component of the Sae system. However, in this study, we have observed an Sae-independent decrease in hemolytic activity following the overexpression of ScrA (Fig. 3C). While a connection between ScrA and the Sae systems still appears likely, this result raises the question of whether ScrA has an additional target (other than Sae) or if the observed increase in Sae activity is a downstream effect of an interaction between ScrA and an as-yet-unidentified protein. Further studies to identify potential interaction partners for ScrA are ongoing in our laboratory and will likely reveal the mechanism behind this regulation.

The results outlined in this study demonstrate that ScrA is a vital component of the S. aureus regulatory network and is required for full virulence in a murine model of infection. We have shown that the abundance of ScrA alters host factor binding and surface protein expression. Additionally, ScrA-mediated hemolysis was shown to be mediated by HlgAB, which is known to be a member of the SaeRS regulon. However, the requirement for Agr and the decrease in hemolysis in the absence of SaeR or SaeS suggest an additional role for ScrA aside from Sae regulation. It is possible that this is due to ScrA activity on an intermediary, which in turn impacts Sae, in addition to as-yet-unidentified targets. However, a possible intermediary has yet to be identified. Regardless, this is suggestive of ScrA acting in a larger regulatory network as opposed to our original model as a direct activator of Sae. In all, this suggests ScrA may play a larger role in S. aureus virulence than originally anticipated.

## MATERIALS AND METHODS

### Strains and strain construction.

All bacterial strains and plasmids used in this study are listed in Table 5. All oligonucleotides are listed in Table 6. Transposon mutants were acquired from the Network on Antimicrobial Resistance in Staphylococcus aureus (NARSA) (49) and transduced into USA300 AH1263. Phage transduction of both transposon mutations and plasmids was performed using bacteriophage Φ11. The presence of a transposon in *scrA* was confirmed by PCR using primer pair 0669/0831. The presence of pMK4 was confirmed by PCR using primer pair 0045/0046.

**TABLE 5:** Strains used in this study

Strain or plasmid	Characteristic	Reference
S. aureus strains		
AH1263	USA300 LAC isolate cured of plasmid LAC-p03	58
RKC0684	AH1263 *saeR*::Bursa	25
RKC0742	AH1263 *scrA*::Bursa	This study
RKC0599	AH1263/pMK4	59
RKC0760	AH1263/pRKC752	25
RKC0763	AH1263 *scrA*::Bursa/pMK4_EV	This study
RKC0878	AH1263 *saeR*::Bursa/pScrAB	25
RKC0908	AH1263 *saeR*::Bursa/pMK4_EV	25
RKC1066	AH1263 *saeS*::Bursa/pScrAB	25
RKC1067	AH1263 *saeS*::Bursa/pMK4_EV	25
RKC0772	AH1263 *agrA*::Ery/pRKC752	25
RKC0694	AH1263 *agrA*::Ery	25
Plasmids		
pMK4	Gram-positive shuttle vector (Cm^r^)	60
pRKC752	pMK4_*scrAB* (vector overexpressing *scrAB* from its native promoter)	25
pRKC1033	pCN51_*scrA* (vector overexpressing *scrA* from a cadmium-inducible promoter)	25

**TABLE 6:** Primers used in this study

Primer	Sequence	Description
0045	GTAAAACGACGGCCAGTG	M13 forward primer
0046	GGAAACAGCTATGACCATG	M13 reverse primer
0669	AAAACTGCAGAAAATTAATGCGATGATTTTTAGC	*scrA* forward primer
0831	CggatccCCTGATAGAATATAATGTACTGTC	*scrA* reverse primer

### Bacterial growth conditions.

S. aureus cultures were routinely grown at 37°C with shaking in tryptic soy broth (TSB). Escherichia coli cultures were grown at 37°C with shaking in lysogeny broth (LB). Where indicated, the following antibiotics were used at the indicated concentrations: chloramphenicol (10 μg/mL), erythromycin (5 μg/mL), lincomycin (25 μg/mL), and ampicillin (100 μg/mL).

### Rotating and static aggregation assays.

Cultures grown overnight were diluted to an OD_600_ of 1, and 1 mL was transferred to a 1.7-mL microcentrifuge tube. Cells were pelleted and washed with phosphate-buffered saline (PBS). Cells were resuspended in 600 μL of either PBS or whole human serum. Cells were incubated at 37°C with rotation for 30 min. Cells were incubated statically at room temperature for 5 min to allow aggregates to settle. The top 100 μL of the culture was removed to a 96-well plate. The cells were resuspended thoroughly by vortexing and then pipetting, and 100 μL was transferred to a 96-well plate. Resuspended cells were incubated at room temperature statically for 40 min. The top 100 μL of the culture was removed to a 96-well plate. The OD_600_ after each incubation was compared to the OD_600_ of the resuspended cells to calculate the percent reduction in the OD_600_.

### Cell wall shaving.

Cell wall shaving was performed as previously described (50, 51). In short, cultures of S. aureus grown overnight were diluted to an OD_600_ of 1, and 1 mL of the culture was resuspended in 500 μL of either PBS or human serum and incubated with rotation at 37°C for 30 min. Suspensions were washed three times with PBS supplemented with 500 μL of 40% sucrose and 20 mM sodium azide. Cells were incubated with immobilized trypsin (catalog number 20230; Thermo Fisher) and suspended in PBS supplemented with 500 μL of 40% sucrose and 20 mM sodium azide at 37°C for 2 h. Cells were pelleted, and the supernatant containing protein fragments was analyzed by mass spectrometry to determine proteins.

### Cell-free human erythrocyte lysis assay.

Hemolysis assays were performed as previously described (25). In short, cultures grown overnight were diluted 1:100 and grown in 25 mL in a 250-mL flask overnight. Cells were pelleted, and the supernatants were filter sterilized. Two hundred microliters of the supernatant was diluted 1:2 with hemolysis buffer (40 mM CaCl_2_ and 1.7% NaCl). Fifty microliters of whole human blood was added to each tube, and the samples were incubated with rotation at 37°C for 10 min. Intact cells were pelleted at 5,000 × *g* for 1 min. One hundred microliters of the supernatant was transferred to a 96-well plate, and the OD_543_ was determined.

### Propidium iodide staining.

Propidium iodide staining was performed as previously described (25). In short, cultures were grown in 5 mL TSB in a 15-mL conical tube overnight. Cultures were diluted to an OD_600_ of 1 and resuspended in PBS. One milliliter of the cells was transferred to a microcentrifuge tube. One hundred microliters was transferred to a white 96-well plate. Propidium iodide was added to the remaining 900 μL, and the samples were allowed to incubate for 5 min at room temperature. One hundred microliters of the stained culture was transferred to a white opaque 96-well plate, and the fluorescence was measured. The fluorescence from the unstained culture was subtracted from the total fluorescence.

### Lipase activity.

The lipase activity assay was modified from a method described previously by Kumar et al. (36). In short, the QuantiChrom lipase assay kit (BioAssay Systems) was used to determine lipase activity according to the manufacturer’s directions. Cultures grown overnight were pelleted, and the supernatant was diluted 1:10 in TSB. Ten microliters of the diluted supernatant was transferred to 96-well plates. The working solution was prepared according to the manufacturer’s instructions and heated to 37°C prior to use. A 140-μL working solution was added to each sample. The OD_412_ was determined at 20 min and 30 min, and the increase in the OD_412_ over this time was used to calculate lipase activity according to the manufacturer’s instructions.

### Mass spectrometry.

Protein concentrations were determined using the 660 protein assay (Pierce). Equal concentrations of protein (200 μg) were processed for liquid chromatography-tandem mass spectrometry (LC-MS/MS) using s-traps (Protifi) (52, 53). Briefly, proteins were reduced with dithiothreitol (DTT), alkylated with iodoacetamide (IAA), acidified using phosphoric acid, and combined with s-trap loading buffer (90% methanol [MeOH], 100 mM triethylammonium bicarbonate (TEAB)). Proteins were loaded onto s-traps, washed, and finally digested with trypsin–Lys-C (Promega) (1:100 [wt/wt] enzyme/protein) overnight at 37°C. Peptides were eluted and dried with a vacuum concentrator. Peptides were resuspended in H_2_O–1% acetonitrile (ACN)–0.1% formic acid for LC-MS/MS analysis.

Peptides were separated using a 75-μm by 50-cm C_18_ reversed-phase high-performance liquid chromatography (HPLC) column (Thermo Scientific) on an Ultimate 3000 ultrahigh-performance liquid chromatography (UHPLC) system (Thermo Scientific) with a 120-min gradient (2 to 32% ACN with 0.1% formic acid) and analyzed on a hybrid quadrupole-Orbitrap instrument (Q Exactive Plus; Thermo Fisher Scientific). Full MS survey scans were acquired at a resolution of 70,000. The top 10 most abundant ions were selected for MS/MS analysis.

Raw data files were processed in MaxQuant (v2.1.4) (https://www.maxquant.org) and searched against the current UniProt S. aureus protein sequence database with the addition of the ScrA protein sequence. Search parameters included the constant modification of cysteine by carbamidomethylation and the variable modifications of methionine oxidation and protein N-terminal acetylation. Proteins were identified using the filtering criterion of a 1% protein and peptide false discovery rate. The protein intensity values were normalized using the MaxQuant LFQ (label-free quantitation) function (54).

Label-free quantitation analysis was performed using Perseus software (v1.6.14.0), developed for the analysis of omics data (55). LFQ intensity values were log_2_ transformed and then filtered to include proteins containing at least 60% valid values (reported LFQ intensities) in at least one experimental group. Finally, the missing values in the filtered data set were replaced using the imputation function in Perseus with default parameters (55). Statistical analyses were carried out using the filtered and imputed protein group files. Statistically significant changes in protein abundances were determined using Welch’s *t* test *P* values and z-scores.

### Murine infections.

A murine model of systemic infection was performed as previously described (56). In short, cultures grown overnight were diluted 1:100 in 10 mL TSB and grown to an OD_600_ of 0.7. Bacteria were resuspended in sterile PBS to a final concentration of 10^7^ CFU/100 μL. Mice were injected retro-orbitally with 100 μL of the bacterial suspension. Infections were allowed to progress for 3 days. Mice were euthanized; the brain, heart, lungs, liver, kidneys, and spleen were harvested; and the organ mass was recorded. Organs were homogenized, serially diluted, and normalized to the organ weight to determine the bacterial burden.

### Ethics statement.

Human blood was obtained according to procedures approved by the Ohio University Institutional Review Board. Blood was obtained from anonymous donors at Ohio University. Animal experiments were performed under the approval of the Institutional Animal Care and Use Committee (reference protocol identifier 17-H-019).

### Data availability.

The mass spectrometry proteomics data have been deposited to the ProteomeXchange Consortium via the PRIDE (57) partner repository with the data set identifier PXD038927.
