# Supplementary material for: The Small Protein ScrA Influences Staphylococcus aureus Virulence-Related Processes via the SaeRS System
Source: Microbiol Spectr. 2023 May 8;11(3):e05255-22. doi: 10.1128/spectrum.05255-22 (PMC10269730; doi:10.1128/spectrum.05255-22)
Supplement: Supplemental file 4 — Fig. S1 and legends of Files S1 to S3. Download spectrum.05255-22-s0004.pdf, PDF file, 4.2 MB [file spectrum.05255-22-s0004.pdf]

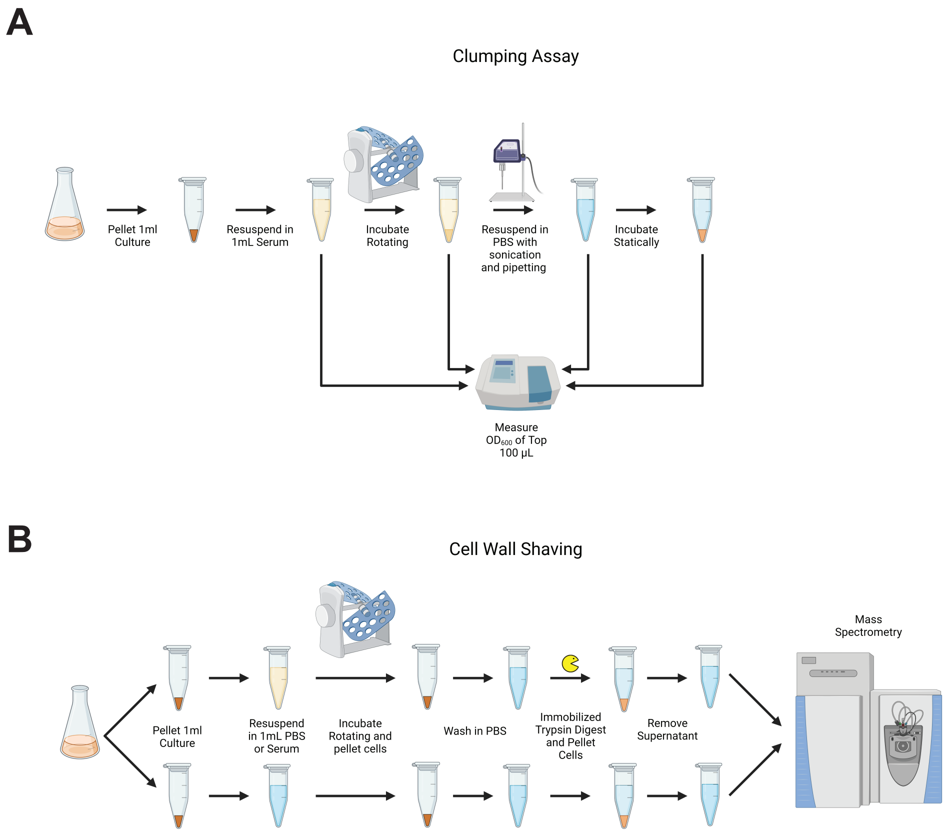

Supplemental Figure S1. Schematic representation of rotating clumping assay (A) and cell wall shaving assay (B)

Supplemental material

Supplemental file S1: Mass spectrometry proteomics showing *S. aureus* proteins identified from samples incubated in PBS.

Supplemental file S2: Mass spectrometry proteomics showing *S. aureus* proteins identified from samples incubated in serum.

Supplemental file S3: Mass spectrometry proteomics showing human proteins identified from samples incubated in serum.
